# Successful resolution of a hemorrhagic pancreatic pseudocyst ruptured into the stomach complicating obstructive pancreatitis due to pancreatic cancer: a case report

**DOI:** 10.1186/s12957-016-0812-x

**Published:** 2016-02-24

**Authors:** Sojun Hoshimoto, Koichi Aiura, Masaya Shito, Toshihiro Kakefuda, Hitoshi Sugiura

**Affiliations:** Department of Surgery, Kawasaki Municipal Hospital, Kawasaki, 210-0013 Kanagawa Japan; Department of Pathology, Kawasaki Municipal Hospital, Kawasaki, 210-0013 Kanagawa Japan

**Keywords:** Obstructive pancreatitis, Pancreatic pseudocyst, Pseudoaneurysm, Pancreatic cancer, Trans-arterial embolization

## Abstract

**Background:**

Hematemesis is uncommon as an initial presenting symptom in pancreatic cancer. We present herein a case of a pseudoaneurysm that ruptured and fistulized into the stomach. The pseudoaneurysm was secondary to a pancreatic pseudocyst complicating obstructive pancreatitis due to pancreatic cancer. The patient was successfully treated using trans-arterial embolization followed by curative surgery.

**Case presentation:**

A 61-year-old man presented to the emergency room with hematemesis. Laboratory examinations revealed a low level of hemoglobin (5.0 g/dl). The patient had presented to another hospital due to hematemesis 1 month before presenting to our hospital. A low-density mass in the pancreatic body with dilatation of the distal main pancreatic duct and a pseudocyst in the pancreatic tail had been observed by radiology at the previous hospital. Further investigation had been planned. Abdominal computed tomography on admission to our hospital demonstrated a pseudoaneurysm in close contact with the wall of the pseudocyst of the pancreatic tail, compressing the stomach. The pseudoaneurysm had not been detected by abdominal computed tomography at the previous hospital. Emergency selective angiography revealed that the pseudoaneurysm arose from the left gastroepiploic artery branching from the splenic artery. Trans-arterial embolization of the left gastroepiploic artery through the splenic artery was successfully performed. Elective distal pancreatectomy and splenectomy with regional lymph node dissection combined with partial resection of the stomach was performed 3 weeks after coil embolization. Pathological examination revealed a moderately differentiated tubular adenocarcinoma in the pancreatic body with regional lymph node metastasis and revealed the pseudoaneurysm rupturing into the pancreatic pseudocyst. The patient has experienced no tumor recurrence or metastasis during 1 year of follow-up.

**Conclusions:**

Spontaneous rupture of a pseudoaneurysm is a rare and potentially lethal complication of a pancreatic pseudocyst. Most affected patients have a history of alcoholism and suffer from acute or chronic pancreatitis. To our knowledge, this is the first reported case of a hemorrhagic pancreatic pseudocyst complicating obstructive pancreatitis due to pancreatic cancer.

## Background

Hemorrhaging is an infrequent complication of pancreatitis but can be lethal, with reported death rates of 6 to 19 % [1–4]. Therefore, spontaneous rupture of a pancreatic pseudocyst into adjacent organs with massive bleeding from a pseudoaneurysm requires rapid management. Most affected patients have a history of alcoholism and suffer from acute or chronic pancreatitis. To our knowledge, spontaneous hemorrhaging of a pancreatic pseudocyst complicating obstructive pancreatitis due to pancreatic cancer has not been described in the literature to date. We present herein the successful resolution of a hemorrhagic pancreatic pseudocyst ruptured into the stomach complicating obstructive pancreatitis due to pancreatic cancer. The patient was treated with trans-arterial embolization (TAE) of the pseudoaneurysm followed by a curative resection for pancreatic cancer.

## Case presentation

**Fig. 1:**
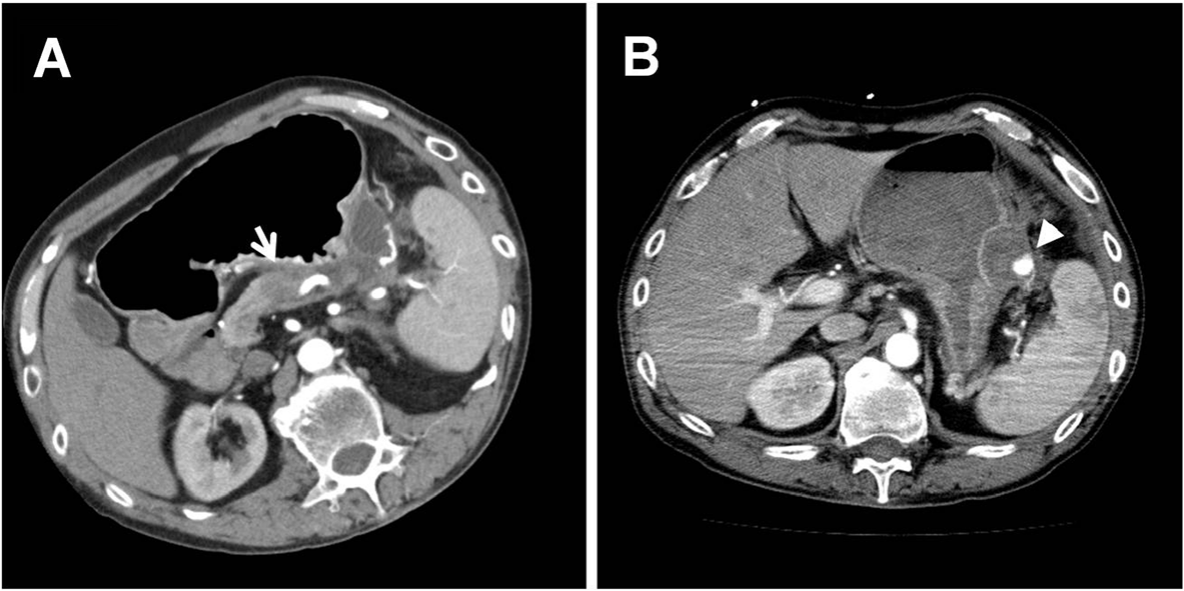
Abdominal computed tomography (CT) of the patient. **a** An initial abdominal CT revealed a low-density mass in the pancreatic body (*arrow*) and a cystic lesion in the pancreatic tail. **b** One month later, a pseudoaneurysm in close contact with the wall of pancreatic pseudocyst was clearly visualized (*arrow head*)

**Fig. 2:**
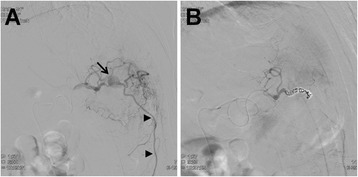
Angiography before and after embolization. **a** Splenic arteriography revealed that the pseudoaneurysm arose from the left gastroepiploic artery branching from the splenic artery. **b** The pseudoaneurysm and the left gastroepiploic artery were successfully embolized using coil embolization

**Fig. 3:**
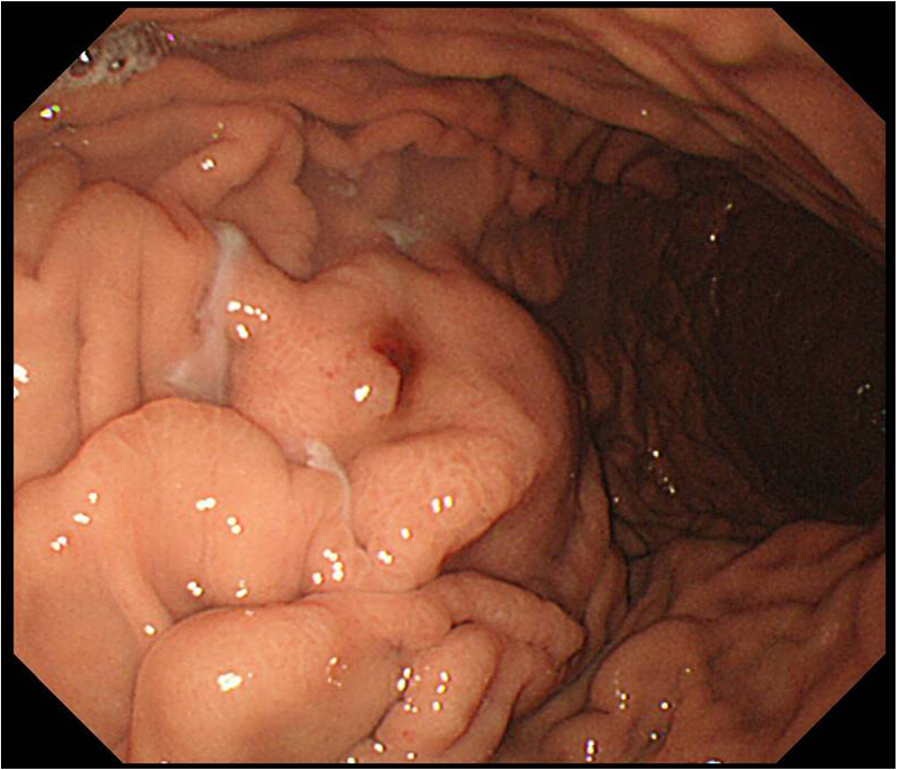
Endoscopic findings. An upper digestive endoscopy the day after trans-arterial embolization revealed an extrinsic compression of the greater curvature in the gastric body with central erosion that appeared to be the fistula orifice between the stomach and the pancreatic pseudocyst

**Fig. 4:**
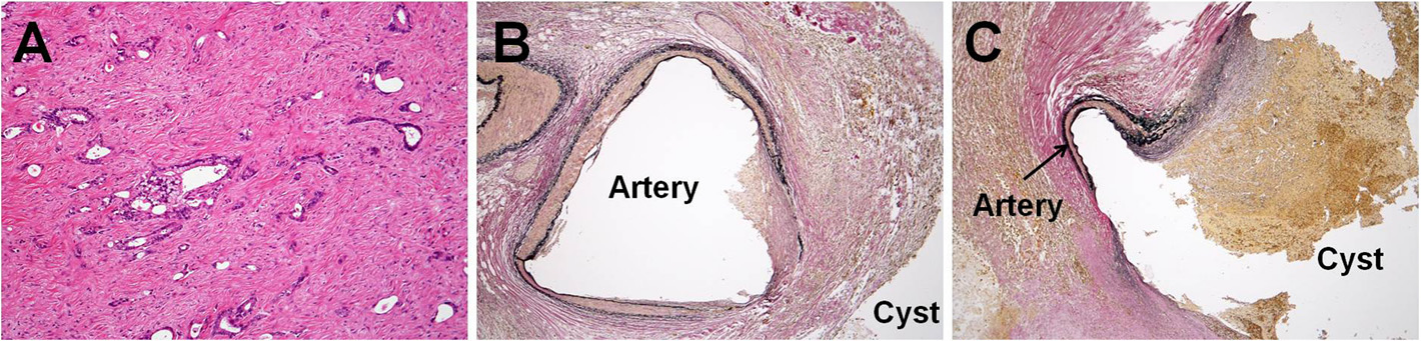
Histopathological findings. **a** Microscopic evaluation after hematoxylin-eosin staining of the tumor tissue revealed moderately differentiated tubular adenocarcinoma (original magnification, ×13). **b** Elastic Van Gieson staining revealed an artery penetrating through the wall of the pancreatic pseudocyst (original magnification, ×6). **c** Elastic Van Gieson staining revealed an artery rupturing into the pancreatic pseudocyst (original magnification, ×6)

A 61-year-old Japanese man presented to the emergency room with hematemesis. He was unconscious due to persistent arterial hypotension. On laboratory examinations, severe anemia was detected (hemoglobin level was 5.0 g/dl). Other laboratory data revealed normal liver, biliary, and pancreatic enzymes. The levels of serum tumor markers, including carcinoembryonic antigen and carbohydrate antigen 19-9, were also within normal limits. Although he had been a habitual alcohol drinker for 40 years, he had no history of pancreatitis or episodes of recurrent abdominal pain. He had presented to another hospital with hematemesis 1 month prior to presenting to our hospital. Abdominal computed tomography (CT) at the previous hospital had revealed a low-density mass of 2.0 cm in size in the pancreatic body with dilatation of the distal main pancreatic duct and a cystic lesion in the pancreatic tail (Fig. 1a). Further investigation had been planned. Abdominal CT on admission to our hospital demonstrated a low-density mass and a pseudoaneurysm in close contact with the wall of the pseudocyst of the pancreatic tail, compressing the stomach (Fig. 1b). The pseudoaneurysm had not been detected by abdominal CT performed at the previous hospital 1 month prior. The pancreatic pseudocyst had increased to 3.8 cm in diameter. The patient was resuscitated after being given a blood transfusion. The lesion was diagnosed as a pseudoaneurysm caused by a pancreatic pseudocyst complicating obstructive pancreatitis due to pancreatic cancer. The lesion had ruptured and fistulized into the stomach. Emergency selective angiography was immediately performed. Splenic arteriography revealed that the pseudoaneurysm arose from the left gastroepiploic artery (LGEA) branching from the splenic artery (Fig. 2a). TAE of the LGEA through the splenic artery was performed. After TAE, complete embolization of the LGEA was confirmed (Fig. 2b). An upper digestive endoscopy the day after TAE revealed an extrinsic compression of the greater curvature of the gastric body with central erosion that appeared to be the fistula orifice between the stomach and the pancreatic pseudocyst (Fig. 3). Bleeding was not observed. Under the diagnosis of pancreatic cancer associated with a ruptured pseudoaneurysm secondary to a pancreatic pseudocyst, elective distal pancreatectomy and splenectomy with regional lymph node dissection combined with partial resection of the stomach was performed 3 weeks after TAE. A firm tumor was located at the body of the pancreas, and the distal pancreas was diffusely hard, which was compatible with pancreatitis caused by obstruction of the main pancreatic duct due to pancreatic cancer. The pancreatic pseudocyst was identified as a firm mass with dense inflammation adhering between the posterior wall of the stomach and the pancreas tail. Macroscopically, the pseudocyst wall consisted of the posterior wall of the stomach and pancreas. The LGEA was located at the wall of the pancreatic pseudocyst. Pathological examination of the tumor tissue revealed a moderately differentiated tubular adenocarcinoma in the pancreatic body with regional lymph node metastasis (Fig. 4a) and the LGEA rupturing into the pancreatic pseudocyst in the pancreatic tail (Fig. 4b, c). In the non-cancerous area of the pancreas, sparse periductal lymphocytic infiltrates and well-preserved lobular structures were observed; however, intralobular fibrosis, duct distortions, protein plugs or calculi, which are all suggestive of chronic pancreatitis, were not observed.

There were no postoperative complications, and the patient has experienced no tumor recurrence or metastasis during 1 year of follow-up.

### Discussion

A pancreatic pseudocyst may hemorrhage into the gastrointestinal tract, peritoneal cavity, retroperitoneum, or simultaneously into more than one of these sites [1, 5, 6]. Cases of spontaneous rupture and fistulization of a pseudoaneurysm into the stomach complicating a pancreatic pseudocyst due to chronic pancreatitis have been reported [7–10]. However, to our knowledge, this is the first reported case of a hemorrhagic pancreatic pseudocyst complicating obstructive pancreatitis due to pancreatic cancer.

Since rupture of a pseudoaneurysm associated with a pancreatic pseudocyst can lead to sudden deterioration of a patient’s condition due to massive bleeding, rapid management is required. In such cases, effective therapeutic procedures include percutaneous, intravascular embolization, or immediate laparotomy [11]. However, laparotomy during hemorrhagic shock can have serious complications. Therefore, TAE is likely to be the first choice for temporary control of bleeding, and urgent surgery should be limited to when embolization fails. Rebleeding has been reported in 37 % of patients, even after successful immediate embolization [11]. Therefore, elective surgery should be carried out after improving conditions by temporary control of bleeding. In the present case, TAE successfully stopped the hemorrhage, allowing curative resection of pancreatic cancer, suggesting that TAE followed by surgery is a beneficial treatment option.

Pancreatic cancer often invades adjacent organs and results in symptoms such as obstructive jaundice, duodenal obstruction, weight loss, and pain. The most common symptom previously reported was hematochezia, even if pancreatic cancer directly invades the gastrointestinal tract. Only a few cases of hematemesis as an initial presenting symptom in pancreatic cancer have been reported [12, 13]. Pancreatic retention cysts with dilatation of the upstream pancreatic duct can occur in pancreatic cancer patients [14]. However, acute pancreatitis is uncommon, and pancreatitis secondary to pancreatic cancer is usually milder than conventional acute pancreatitis [15, 16]. In the present case, no pseudoaneurysm was observed by abdominal CT during the initial hematemesis episode. However, a pseudoaneurysm in close contact with the wall of a pancreatic pseudocyst was clearly visualized on abdominal CT performed 1 month later during a second hematemesis episode, suggesting that erosion of the left gastropiploic artery developed in 1 month, despite no symptoms during this period.

## Conclusions

To our knowledge, this is the first reported case of a hemorrhagic pancreatic pseudocyst that ruptured into the stomach, complicating obstructive pancreatitis due to pancreatic cancer. The patient was successfully treated with TAE followed by curative surgery. TAE may be indicated to immediately stop a hemorrhage, allowing greater safety during the subsequent curative surgery in this uncommon situation.

### Consent

Written informed consent was obtained from the patient to allow publication of this case report and any accompanying images. A copy of the written consent is available for review by the Editor-in-Chief of this journal.

## Abbreviations

CT: computed tomography
LGEA: left gastroepiploic artery
TAE: trans-arterial embolization
